# A Course of Clinical Lectures, Delivered at the Hôtel-Dieu, Paris, for the Session 1842-’43

**Published:** 1843-02-18

**Authors:** A. F. Chomel

**Affiliations:** Paris


### THE MEDICAL EXAMINER,

### And Retrospect of the Medical Sciences.

Vol. VI.] PHILADELPHIA, SATURDAY, FEBRUARY 18, 1843. [No. 3.

### A COURSE OF CLINICAL LECTURES,

*Delivered at the Hôtel Dieu, Paris, for the Session* 1842–'43.

BY A. F. CHOMEL, M. D.

### LECTURE II.

### ON HEPATIC COLIC.

Gentlemen,—At No. 1, of the Salle St. Bernard, is a woman to whom I have called your attention, for the past few days. She has been a prey to the most horrible acute attacks, and her sufferings, at times, have been so intense, as to prevent her maintaining any fixed position. During the paroxysm her whole body is in a state of perpetual spasm; sometimes she lies on her side, and sometimes on her back, without being able to procure any relief from her sufferings by position. Her expression too is much changed, and denotes very plainly her intense suffering. To this state of excitement calm succeeds, and at the morning's visit, you can hardly believe, if you had not been witness of the scene, that this patient had suffered these tortures the evening previous. I shall this morning enter into some details respecting this case. And, let us first inquire into the cause of these acute sufferings: Was it an inflammatory affection of the intestinal canal, or of the peritoneum? This hypothesis cannot be admitted, as we have seen the patient lie on her belly, and compress strongly this region with her hands, without any augmentation of pain; on the contrary, it seemed to give her some relief. Now if the intestine, or its serous covering, had been the seat of phlogosis, all the movements which this patient made could only have exasperated the pain. We must give up all idea of inflammation in the organs in question. Whenever we see a patient a prey to intense abdominal pain, able to lie on his belly, and compress it with impunity, and even with relief, you may be pretty certain there is no inflammation there. Hence we must seek elsewhere the cause of suffering. This is not the first time, it seems, that this patient has experienced similar attacks. Seven or eight days since the same state of things occurred; the pain seemed to have, as now, the same point of departure—the epigastric region—irradiating itself from thence towards the sides, and fixing itself principally in the hypochondriac region. Then too, as now, the attack commenced in the evening, and after having lasted all night, ceased towards morning under the influence of opium. Similar scenes have been acted five times in the space of ten days, leaving each time some tenderness of the belly, and some general prostration; but without having been accompanied with vomiting, without diarrhœa or fever; the pulse, on the contrary, being rather below the natural standard. Here too is additional and positive proof that we have nothing to do with an acute inflammation of the principal abdominal organs. What viscus then is the seat or the point of departure of these pains? There are several organs which are capable of presenting a similar collection of symptoms; the stomach for example, becomes the seat of intense pain after the reception of a substance which disagrees with it; but in this case, which is no other than an attack of indigestion, there is always vomiting of the indigestible matter, and the symptoms once dissipated, do not return. Now nothing of this kind has taken place in our patient; she has taken nothing indigestible; all her food having been some light soup; she has not vomited; and the same train of symptoms have occurred several times, with intervals of calm of some length.

Hysteria, that proteiform malady, can, in some women, produce very severe suffering, the pain having its principal seat in the epigastric region. But in general these pains appertain to individual idiosyncrasy; a moral cause may be recognized as their origin. Hence those that we commonly see thus affected are fashionable women, of a highly nervous temperament, of lively passions, and ardent imagination; or certain women of the lower class, who, by position or profession, are in intercourse with the higher classes, from whom they borrow, so to speak, their moral character. Our patient belongs to neither of these classes; she has never suffered from hysteria. Before her entrance into the hospital, she had had a post-puerperal metritis. The first time that I saw her, the uterus was somewhat enlarged, and some sensibility persisted in the hypogastric region. But all these symptoms disappeared in a little time.—There was pain produced either by pressure on the lower part of the belly, and by an examination *per vaginam.* Besides, had the epigastric pains she has lately experienced, proceeded from the uterus, they would have been of entirely a different character. We cannot, therefore, look to this cause to explain the etiology and seat of the disease which occupies us. Sometimes rheumatism can affect isolated organs; leaving, in fact, the surface, to fix itself, by a genuine metastasis, upon some internal organ, where it ordinarily but rarely appears—as the stomach, intestines, bladder, &c. In these cases the pain is very acute, but it is never primary; the patient having suffered previously from rheumatism in some external organ, or their parents having been similarly affected. Besides, rheumatismal pain never reaches the same degree of intensity, and wants the distinctive character which the sufferings of our patient presents. The periodical return of the pain towards evening, its cessation in the morning, with the intervals of entire calm, might, for a moment, lead us to think of a class of intermittent affections called larvated fevers. We have hence inquired of the patient whether she has ever suffered from any disease of this kind, or ever inhabited any place where it prevailed, and she has answered in the negative. Essential intermittent fevers, too, present characters entirely different. In them the access takes place generally during the day; it is always preceded by a chill, followed by heat, and perspiration, more or less profuse. In our patient the access occurs ordinarily in the evening, is prolonged through the night without shivering, or any change in the temperature of the skin, for, as I have already remarked, the pulse has never presented a febrile character. Let us add, finally, that the attacks have always been mitigated by the use of opium, or similar preparations. Now, intermittent fever rarely yields to opium. Hence we must abandon that idea, and look elsewhere for the cause. Lead and its preparations sometimes cause very severe pain in persons exposed to their influence. Saturnine colic offers nocturnal exacerbations rather than diurnal. But in this case the cause has not existed, or, to speak more strictly, to a sufficient degree; the patient tells us indeed that she was for sometime in the service of a dealer in paints, but she left it some four or five months since, and has never, in fact, experienced any symptoms which might be properly referred to a saturnine affection.

All these causes being eliminated, there remains only as the probable cause of the disease which occupies us, a material cause, such as the presence of a calculus, either in the ureter, constituting nephritic colic; or in the ductus choledochus, hepatic colic; or, the existence of a pancreatic calculus. This last affection, although very rare, has occasionally been successfully diagnosticated. In a woman, who died in this hospital soon after entrance, with symptoms referable to an affection of this nature, I found the pancreatic canal obliterated; all the pancreatic ducts were of the size of a goose's quill; they were united together, agglomerated, and resembled in appearance the gall-bladder. The proper tissue of the pancreas had nearly disappeared, and there remained only the internal and external membranes, the parenchyma being replaced by this collection of enlarged ducts. The alteration of tissue was similar to that which you observe sometimes in the kidneys, when the tubuli uriniferi and the pelvis become so much dilated, that they occupy the entire thickness of the organ, which is reduced to its enveloping and lining membranes, its cortical substance having entirely disappeared. I mention this fact to you in order that you may continue to collect observations in connection with the subject, for the number of registered facts of this kind are small, and quite insufficient to enlighten this point of pathological anatomy. This cause too I may then set aside as improbable. There remains, therefore, an hepatic or a nephritic calculus to account for the production of these symptoms. I do not think that there is any question of the latter affection, because the patient continues to urinate well, and we have never discovered any trace of blood or of calculous concretions in her urine. Besides, she has never complained of pain in the lumbar region, or along the course of the ureters, nor in the bladder; all which are constant symptoms in nephritic calculus. In fact, it is the clear and characteristic train of symptoms which renders the diagnosis of nephritic calculus so easy, indeed so much more easy than that of hepatic colic. Hence it is that we arrive at a correct diagnosis of this latter disease rather by the method of exclusion, than by any direct means.

Hepatic colic is a very interesting disease to study. It results from the passage of a biliary calculus from the gall-bladder by the ductus choledochus communis. I say a calculus proceeding from the gall-bladder, for it is there that these bodies are formed, and never elsewhere. So long as these bodies remain in the gall-bladder they occasion no pain, but the moment they leave it, and enter the ductus choledochus, the compression as well as the laceration of that canal which they produce, cause most excruciating agonies. It is not necessary for pain to be produced that the caliber of the canal be entirely filled up by the calculus, it is sufficient, as is commonly the case, that the gall-stone be angular, rough, and capable of lacerating the parietes of the canal, permitting at the same time a certain quantity of bile to escape past the inequalities. A calculus thus engaged in the duct is often capable of producing jaundice, but this symptom is not a necessary consequence of the presence of a calculus in the gall-duct, as some physicians have advanced. I have seen, and others, likewise, patients affected with hepatic colic, without any jaundiced tint. A further proof that this accident is not necessarily connected with the presence of biliary calculi, is the instability of the yellow colour in this class of patients. It appears and disappears rapidly from one day to another. Now this state of things does not occur in genuine icterus. The yellow hue, in this disease, persists ordinarily five or six weeks; it is the phenomenon in jaundice which lasts the longest; it sometimes remains when all has returned to a natural condition. A patient now in the wards presents an example of the transient nature of icterus produced by hepatic colic. I shall revert to this point subsequently. Hence we see that the absence of a yellow hue of the skin should not make us exclude the idea of the existence of hepatic colic. Thus two days since when I first called your attention to this patient, without saying anything of the cause or nature of the severe pain from which she was suffering, I should have been able then to have diagnosticated, by that symptom alone, an hepatic colic, in spite of the absence of jaundice. To day this symptom has appeared; her urine is of a deep yellow colour; there is hence no longer any doubt in this respect.

Some physicians think that when there exists pain, which appears and disappears, alternating with intervals of calm without vomiting, you may pretty surely suspect, though you cannot safely diagnosticate, hepatic colic. You should know that some patients experience no other symptom than acute pain, without vomiting, and without the rejection of the gall-stone by the mouth, or its passage by stool, and this is fully explained. When a calculus is arrested in the biliary canal, the hepatic secretion augments, and in consequence dilates by degrees that portion of the canal which is below the point occupied by the gall-stone, at the same time that the superior portion becomes constricted. It hence results that the stone not being able to advance on account of the narrowing of the canal, remains where it was stopped, or going backwards, returns into the gall-bladder. It is thus that we explain these attacks of colic unaccompanied by vomiting, or by the rejection of the biliary secretions. At the end of some time some fragments of calculus escape through a strictured portion of the canal, pass into the stomach or the intestines, and thence is rejected by vomiting, or voided in the stools, in the midst of a mass of excrementitious matter, where they may pass unnoticed. It may happen that the gall-stones may not be rejected externally, that they may have retraced their way, but they become dissolved in situ, without producing any of those symptoms which characterise, according to certain physicians, the disease of which I am treating. To day we have no longer any doubt on the score of our patient; we have as complement the symptom of jaundice well pronounced. Hence the real cause of the sufferings of this patient is the presence of biliary calculi in the ductus choledochus communis.

The study of these affections has been very much neglected for some time; it is, however, a subject of the highest importance. I cannot too much impress on you the frequency of this disease, or the number of individuals who are a prey to these intense sufferings from this cause.

At the time when the physiological doctrine was in all its vigor, hepatic colic was attributed to an inflammatory condition of the liver, and it was treated by purely antiphlogistic means. Now adhering to the close observation of facts, you will find that rarely, if ever, a genuine inflammation of that organ ever caused any obstruction to the passage of the bile along the ductus choledochus. Dr. Andral himself, who published one or two facts apparently confirming the ideas of Broussais in this respect, acknowledged subsequently, with that frankness which characterises him, that he was influenced by the peculiar tone of the medical epoch in his interpretation of the phenomena. Genuine hepatitis, therefore, never produces hepatic colic, such as we are observing. Remark, in fact, what occurs in the various inflammations of the buccal cavity. Do you ever see as the consequence of these inflammations, accompanied often by the production of false membranes which line the entire mouth,—do you ever see, I say, the obliteration of the duct of Steno, or the ducts of Wharton, and the suppression of the flow of the saliva? On the contrary, the secretion ordinarily increases, and the flow of the secreted fluid is very free. The theory of Broussais was therefore pure hypothesis, which could not subsist in the face of facts and of reason.

What are then the causes of this disease? They are, we must acknowledge, very obscure. Veterinary medicine may spread some light on the subject. Veterinary surgeons have remarked that animals slaughtered during winter, after having been stall-fed on dry vegetables and other tonic substances, and kept without exercise, frequently present a large number of biliary calculi in the gall-bladder and bile ducts; whilst, on the contrary, these products are rare in animals killed in the spring and autumn, and who have been fed on fresh vegetable diet, and have lived in the open air. Hence, from analogy, it results that in man also, a sedentary life, a stimulating animal diet, and the privation of fresh vegetable food, contribute powerfully to the development of biliary calculi, and to the production of hepatic colic. From these facts we may deduce, too, the hygienic treatment adapted to these affections. Fresh vegetable diet, moderate exercise, and the use of alkaline drinks, such as the Vichy water, etc., to which we may add, for the medical treatment, purgatives in small and repeated doses, in order to facilitate the expulsion of the calculi which occupy the gall-duct. To effect this we should augment the biliary secretion, which mechanically forces the passage of the gall-stones arrested in the canal; this is the indication for purgatives. Turpentine and ether may be rubbed on the epigastric region, and over the liver, and you may even administer them together after the formula of Durand, who first proposed this treatment, and attributes much success to it. It is by this and other similar means, which you will find described in the special treatises, that you must combat this disease in the intervals of calm. During the attack you must assuage the sufferings of your patient by the use of opium, which you should administer in increasing doses, commencing with one grain, and sometimes reaching the quantity of fifteen to twenty grains in the course of the day; this quantity, of course, being given in fractional doses every hour, until some relief be obtained, or until some contra-indication occur. You should, however, watch closely the effects of this powerful remedy. When it is not borne by the stomach, you can use it by the rectum. This mode of administration does not demand less care and watching than the former; for you can as easily narcotise your patient in this manner as by the stomach, absorption being very active. When the attack persists for a long time the liver becomes enormously distended, and very sensible; fever occurs, and true inflammation sets in; you must then act accordingly; you must combat this secondary complication by local and general antiphlogistics, more or less energetic.

This disease, very painful for the patient, and alarming to the attendants, is rarely fatal; much less so, indeed, than nephritic colic. But you should be told that a radical cure is very difficult to effect. After the lapse of some time the ductus choledochus, where the calculus is stopped, dilates, and leaves a free passage to the foreign body, which is expelled either by the stomach or the rectum. At other times, as I remarked before, the canal becomes obstructed, the calculus becomes encysted, as it were, and disappears by degrees by absorption. Sometimes, in consequence of the entire obliteration of the canal, there is an arrest of the bile, which finishes by producing those serious disorders in the biliary apparatus which end in death.

At the autopsy of these cases you find the biliary ducts enormously distended at their superior portion whilst they are very much contracted at the inferior part; the gall-bladder is excessively dilated; the liver is swollen and filled with bile. In some rare cases you find profound organic alterations, and the biliary ducts filled with pus. Such are very nearly the lesions which you meet with in the very rare cases of fatal termination from this disease. This disease, I repeat, is very painful, but rarely followed by death.

*Paris, Dec.* 1842.

